# Understanding the Loss of Maternal Care in Avian Brood Parasites Using Preoptic Area Transcriptome Comparisons in Brood Parasitic and Non-parasitic Blackbirds

**DOI:** 10.1534/g3.118.200992

**Published:** 2019-02-13

**Authors:** Kathleen S. Lynch, Lauren A. O’Connell, Matthew I. M. Louder, Christopher N. Balakrishnan, Eva K. Fischer

**Affiliations:** * Department of Biology, Hofstra University, Hempstead, NY 11549; † Department of Biology, Stanford University, Stanford, CA 94305; ‡ Department of Biology, East Carolina University: Greenville, NC, 27858; § Department of Animal Biology, University of Illinois at Urbana-Champaign, Urbana, IL 61801

**Keywords:** brood parasitism, maternal behavior, preoptic area, transcriptome, neoteny

## Abstract

Parental care is critical for offspring survival in many species. However, parental behaviors have been lost in roughly 1% of avian species known as the obligate brood parasites. To shed light on molecular and neurobiological mechanisms mediating brood parasitic behavior, we compared brain gene expression patterns between two brood parasitic species and one closely related non-parasitic Icterid (blackbird) species. Our analyses focused on gene expression changes specifically in the preoptic area (POA), a brain region known to play a critical role in parental behavior across vertebrates. Using comparative transcriptomic approaches, we identified gene expression patterns associated with brood parasitism. We evaluated three non-mutually exclusive alternatives for the evolution of brood parasitism: (1) retention of juvenile-like (neotenic) gene expression, (2) reduced expression of maternal care-related genes in the POA, and/or (3) increased expression of genes inhibiting maternal care. We find evidence for neotenic expression patterns in both species of parasitic cowbirds as compared to maternal, non-parasites. In addition, we observed differential expression in a number of genes with previously established roles in mediating maternal care. Together, these results provide the first insight into transcriptomic and genetic mechanisms underlying the loss of maternal behavior in avian brood parasites.

Parental care has evolved repeatedly and independently across taxa, likely because it increases offspring survival in the face of environmental pressures such as high predation or unpredictable access to resources (Clutton-Brock 1991;Klug and Bonsall 2010; Caron and Vannier 2016). Broadly defined, parental care is any behavior on the part of parents that increases the survival and fitness of their offspring (Buntin 1996; Numan and Insel 2006; Adkins-Regan 2013). Parental behaviors are varied, but include behaviors such as nest building, defense, cleaning/grooming, and provisioning of offspring. While parental care improves offspring survival it comes at a cost to the individuals providing the care. Direct costs include increased predation risk and decreased foraging time and indirect costs include reduction in short- and long-term reproductive output (Clutton-Brock 1991; Alonso-Alvarez and Velando 2012). The costs and benefits of performing parental care likely contributed to the evolution of diverse forms of parental care within and between taxa, as well as between individuals and sexes (Clutton-Brock 1991; Klug *et al.* 2013). Remarkable divergence in the magnitude of parental care within and between species provides fertile ground to study this behavior. Specifically, animals that use an evolutionarily derived parental care strategy, rather than a strategy ancestral to their group, may provide unique insight into the genetic architecture of parental care.

In birds, offspring care occurs in many forms including bi-parental care, uniparental care, and forms of cooperative breeding where siblings provide care (Lack 1968; Cockburn 1998; Cockburn 2006). However, roughly 1% of the approximately 10,000 described species of birds are obligate brood parasitic, an evolutionary derived strategy in which males and females display no parental care (Winfree 1999). Obligate brood parasites have completely lost parental behaviors including constructing nests, incubating eggs, and provisioning their young. Instead, they leave their eggs in the nest of another species, often with considerable negative impacts on the reproductive success of the host species. Consequently, avian brood parasites reap the benefits of parental care without the costs. Evolutionary losses in avian parental behavior have occurred seven independent times with three origins among cuckoos, and one origin each in cowbirds, honeyguides, Old World finches, and a South American duck (Lanyon 1992; Davies 2000; Sorenson and Payne 2002). Whereas, the other six origins of brood parasitism occurred over ∼15 million years ago (Barker *et al.* 2013), the Molothrus genus (family *Icteridea*) represents the most recent transition to brood parasitism. Non-parasitic and parasitic Icterids are estimated to have diverged roughly ∼5 million years ago (Barker *et al.* 2013).

Behavioral ecologists have provided multiple excellent explanations for the evolution of brood parasitism including extreme limitation of nesting sites and/or dilution of the negative impacts of nest predators by not putting all one’s eggs in a single nest (Winfree 1999; Davies *et al.* 1989; Hamilton and Orians 1965; Payne 1977; Rothstein 1993; Semel and Sherman 2001). On the other hand, genetic and neurobiological perspectives for the appearance of avian brood parasitism are entirely lacking. The bulk of what is known about the neurobiological basis of brood parasitic behavior comes from a single published abstract which presented a study of region-specific prolactin receptor abundance (Ball 1991). Prolactin receptor abundance was quantified in the preoptic area (POA), a region central to maternal behaviors across all vertebrates that display parental behavior (Numan and Insel 2006; Adkins-Regan 2013; Chokchaloemwong *et al.* 2013; Rivas *et al.* 2016). The POA is rich in steroid, peptide, and neurotransmitter receptors, all of which modulate maternal behavior (Numan and Insel 2006). In brown-headed cowbirds (*Molothrus ater*), an obligate brood parasite ubiquitous across North America, prolactin binding sites within the POA exhibit reduced sensitivity (Ball 1991) as compared to red-winged blackbirds (*Agelaius phoeniceus*), a closely related blackbird that is not brood parasitic (Figure 1A). Ball (1991) thus implicated modification of POA gene expression as a neural mechanism mediating the evolutionary transition to brood parasitic behavior; however, this hypothesis remains untested.

**Figure 1:**
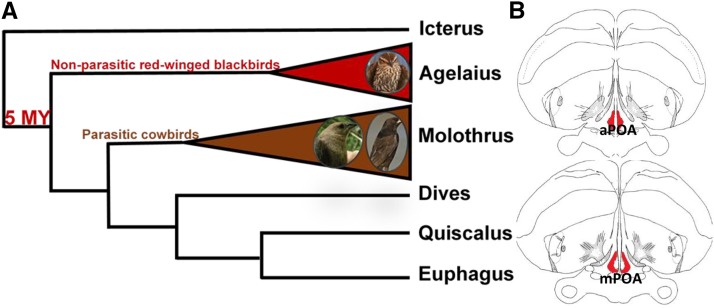
Overview of the Icterid genera (*i.e.*, blackbirds) and target brain regions. (A) Blackbird phylogeny including the three focal species: red-winged blackbirds (*Agelaius phoeniceus*), bronzed cowbirds (*Molothrus aeneus*) and brown-headed cowbirds (*Molothrus ater*). The brown branch of the phylogeny represents the only brood parasitic genus within the Icteridae family. Non-parasitic and parasitic Icterids are estimated to have diverged roughly ∼5 million years ago (Barker *et al.*, 2013). (B) Illustration of the blackbird brain with the preoptic area (POA) collected for transcriptome assembly and gene expression analysis in red. Contained within the POA are the anterior (aPOA) and medial (mPOA) subdivisions. We collapsed these regions into a single sample for all procedures and analyses.

Here, we explore the evolution of brood parasitism using POA-specific gene expression comparisons. We take advantage of divergence in maternal care within North American Icterids (*i.e.*, blackbirds) to identify POA-specific gene expression differences associated with obligate brood parasitism at the transcriptomic level. We focused our analyses specifically on the POA as this brain region is critical for regulation of parental care in male and female vertebrates and was previously implicated in the transition to brood parasitic behavior. By comparing POA gene expression at the transcriptomic level, we identify suites of genes associated with brood parasitic behavior and assess alternative hypotheses regarding the evolutionary origins of this behavior. We complement broad scale analyses with targeted exploration of expression changes in candidate genes known to be important in maternal care.

We compare two brood parasitic Icterids, brown-headed (*Molothrus ater*) and bronzed cowbirds (*Molothrus aeneus*), with juvenile and adult red-winged blackbirds (*Agelaius phoeniceus*), a non-parasitic Icterid. Our aims are threefold: (1) identify transcripts in the POA that are differentially expressed in both brood parasitic species as compared to the maternal non-parasitic species, (2) characterize expression patterns specifically in genes with known roles in social behavior and maternal care and, (3) ask whether expression patterns of these genes in parasites resemble juvenile or adult expression in non-parasites to test the prediction that brood parasitic behavior is mediated by a ‘failure’ to transition to a reproductively mature maternal state. Such a juvenile-like pattern in gene expression would suggest that brood parasites retain neotenic gene expression patterns in the POA. Neoteny is a developmental pattern that occurs when adults retain juvenile characteristics, and therefore does not require novel mechanisms or genetic variants, but rather relies on the alteration of existing mechanisms via shifts in developmental timing. Here, we examine the compelling hypothesis that whole suites of neotenic traits are underpinned by broad-scale neoteny in gene expression.

## Materials and Methods

### Bird capture and treatments

All procedures listed here were reviewed and permitted by Hofstra University IACUC (#13/14-18). Female brown-headed (N = 14) and bronzed cowbirds (N = 17) as well as adult female red-winged blackbirds (N = 4) and recently fledged female red-winged blackbirds (N = 5) were captured using mist nets (bronzed cowbirds and red-winged blackbirds) and bait traps (brown-headed cowbirds) in May-June of 2014 and 2015 in Travis and Hildago counties of Texas. Smaller sample sizes in red-winged blackbirds were due to the lower population densities and capture rates. We used only females in this study because male red-winged blackbirds vary considerably in the amount and quality of care they provide to offspring (Muldal *et al.* 1986). All birds were transported to Hofstra University and housed in outdoor aviaries for at least two weeks and fed a modified Bronx Zoo diet with mealworm supplements. Total time in captivity was approximately one month. This amount of time in captivity decreased reproductive behaviors (*i.e.*, egg laying) in all subjects and homogenized the reproductive states of the females, which allowed maternal hormones to be regulated exogenously. To deliver hormones, we implanted osmotic minipumps as described in Buntin and Buntin 2014; Buntin *et al.* 1999; Wang and Buntin, 1999. Briefly, females received subcutaneous osmotic pumps implants releasing 0.5µl of ovine prolactin / hour for 7 days or vehicle alone (brown-headed cowbirds: N = 9 prolactin, N = 5 vehicle; bronzed cowbirds: N = 11 prolactin, N = 6 vehicle; adult red-winged: N = 4 prolactin; juvenile red-winged N = 5 prolactin; osmotic minipump: Azlet, model 1007D, DURECT Corp. Cupertino, Ca; ovine prolactin: Sigma; St. Louis, Mo.; 3.3µg/hr; 80 µg/day in 0.87% NaCl/0.01M NaHCO3, 3:1 ratio v/v: 12µl/day or sodium bicarbonate vehicle alone). Females were rapidly decapitated and brains flash frozen on dry ice. However, results ultimately revealed that only three transcripts differed between treated and untreated bronzed cowbirds and only a single transcript differed between treated and untreated brown-headed cowbirds. Thus, very few transcriptome differences occurred in the brood parasitic species due to prolactin treatments (Table S1). Given the lack of differences in POA gene expression following prolactin treatment, we combined treated and untreated birds in all further analyses. Effects due to captivity and implants were minimized by uniform captivity duration and the use of control (*i.e.*, vehicle only) implants.

### Sectioning and sequencing

Brain tissue was sectioned into 200µm coronal sections on a Leica CM1950 cryostat and thaw mounted onto microscope slides. Contained within the POA are the anterior (aPOA) and medial (mPOM) subdivisions (Figure 1B). We collected tissue from both regions and pooled it into a single sample that we collectively refer to as the POA. After confirming POA boundaries (Figure 1B), a 1.22 mm diameter tissue punch (Myneurolab, Leica, Richmond IL) was isolated and placed into 500µl of Trizol (Fischer Sci., Waltham, Ma.). POA sections from both hemispheres were collapsed into one tube. All POA sections along the midline to the third ventricle from the caudal split in the tractoseptomesenphalicus to the rostral anterior commissure were collected. RNA was isolated following Trizol manufacturer specifications and was immediately followed by mRNA isolation using NEXTflex PolyA Beads according to manufacturer specifications. RNA sequencing library prep was completed using NEXTflex Rapid Directional RNA-seq based kits. Briefly, first strand synthesis was completed via RNA fragmentation immediately followed by NEXTflex rapid reverse transcriptase. The assembled product was placed in a NEXTflex directional second strand synthesis and immediately cleaned up using Agencourt AMPure XP beads. NEXTflex adenylation mix was used for end repair on second strand synthesis DNA. Adaptor ligation was completed using NEXT flex ligation mix and NEXTflex RNAseq Barcode Adaptors immediately followed by another cleanup with AMPure XP beads. Cleaned up DNA was combined with NEXT flex Uracil DNA Glycoslylase, NEXTflex PCR Master Mix and NEXTflex Primer Mix and amplified using standard PCR. PCR product was immediately cleaned up using AMPure XP beads. Sample libraries were sequenced into 100 base pair paired-end reads at Harvard University using an Illumina HiSeq 2500 following procedures described in (Ewing and Green 1998; Lunter and Goodson 2011).

### Transcriptome construction and annotation

*De novo* transcriptomes were constructed for each species separately. Rcorrector amended Illumina sequencing errors (Song and Florea 2015) and reads were trimmed using Trim Galore! (Babraham Bioinformatics, Babraham Institute) to remove Illumina adapters and restrict all reads to only high-quality sequence. Following developer recommendations, a quality score of 33, a stringency of 5, and a minimum read length of 36 bp were used. Corrected trimmed reads from all individuals by species prior to transcriptome construction were pooled and Trinity (Grabherr *et al.* 2011; Haas *et al.* 2013) was used to construct reference transcriptomes for each species. Initial assemblies contained 296,578 contigs for brown-headed cowbirds, 394,219 contigs for bronzed cowbirds, and 243,189 contigs for red-winged blackbirds.

Raw transcriptome assemblies were filtered using several approaches. CD-HIT-EST (Weizhong Li Lab) was used to cluster overlapping contigs and removed any contigs that were smaller than 250bp following clustering. To remove contaminant sequences, we annotated sequences using blastx queries against the SwissProt database and retained only those contigs with annotations to known vertebrate genes. Default parameters for blastx queries with an e-value cutoff of 10^−10^ were used because this minimized contaminants while allowing a focus on known vertebrate genes. Final assembly annotation was done using Trinotate (Bryant *et al.* 2017) and assembly completeness was assessed using BUSCO (Simão *et al.* 2015) based on conserved ortholog content across highly conserved vertebrate genes. Assembly completeness estimates were 64% in brown-headed cowbirds, 78% in bronze cowbirds, and 50% in red-winged blackbirds, which are in accordance with expectations for single brain region transcriptomes (MacManes 2016). All high-powered computing for transcriptome assembly and filtering was performed on the Odyssey computer cluster supported by the FAS Science Division Research Computing Group at Harvard University.

### Identification of orthologs, read quantification, and differential expression analysis

A matched set of orthologs found in each of the three transcriptome assemblies was generated to compare expression across species in an unbiased way. Open-reading frames were aligned to identify protein-coding genes and to ensure high-quality sequence alignment. TransDecoder predicted the ORFs for each contig in each species-specific final transcriptome (Haas *et al.* 2013). The resulting ORF sequences were then clustered and reduced using CD-HIT (%ID = 99.5). We then used the longest ORF for a given ‘Trinity gene’ and identified orthologs present among all three species with a reciprocal best hit blast approach using blastp with an e-value cutoff of 1e^-20^ (Moreno-Hagelsieb and Latimer 2008). The predicted polypeptides for orthologs were then aligned with MAFFT (Katoh and Standley 2013) and the corresponding coding sequences were back-aligned with pal2nal (Suyama *et al.* 2006). Finally, each alignment was subjected to alignment scoring and masking using ALISCORE (default parameters) and poorly aligned regions were trimmed using ALICUT (Kück 2009). This procedure left 12,237 aligned orthologs shared across all species. Complete assemblies were filtered with this list to obtain species specific transcriptomes that contained only transcripts that were matched and aligned across the three species.

Reads were mapped and read abundance estimated with Kallisto (Bray *et al.* 2016) using default parameters. Read counts were combined into a single matrix for all individuals from all species based on ortholog identification and this count matrix was used for all downstream analyses.

To characterize expression variation among all orthologous transcripts, we first ran principal components analysis. We used ANOVAs to test for group differences in principal component (PC) scores. We tested for group differences based on variety of factors, including species, prolactin treatment, age (*i.e.*, adult *vs.* juvenile), reproductive strategy (*i.e.*, parasites *vs.* non-parasites), and various combinations of these factors. Principal component analysis and ANOVAs were performed in R (version 3.3.2; The R Foundation for Statistical Computing).

We tested for differential expression (DE) of orthologs using DESeq2 (Love *et al.* 2014) to run standard, pairwise DE analysis for our comparisons of interest. We corrected p-values for multiple hypothesis testing using standard FDR correction and set our adjusted p-value cutoff to <0.05. We identified contigs DE in the following four comparisons: (1) each brood parasite compared to adult non-parasite (*i.e.*, bronzed cowbird *vs.* red-winged and brown-headed cowbird *vs.* red-winged), (2) between brood parasite species (*i.e.*, bronzed *vs.* brown-headed cowbirds) and, (3) between adult and juvenile non-parasites. Following DE analysis, we performed GO term enrichment analysis for DE genes using the ‘Biological Processes’ GO categories in the topGO package (Alexa and Rahnenfuhrer 2009) in R (version 3.3.2; The R Foundation for Statistical Computing).

After initial exploration of our data, we hypothesized that brood parasitism may reflect an absence of the transition to maternal patterns of gene expression in the parasite POA, such that expression remains juvenile-like (neotenic). To address this possibility, we asked whether expression changes between parasitic cowbird adults and non-parasitic blackbird adults mirrored those between juvenile and adult red-winged blackbirds. We first compared POA gene expression in red-winged blackbird juveniles and adults. We were able to use the direction of expression differences from this comparison to assess whether expression patterns in cowbirds were more juvenile-like (neotenic) or adult-like as compared to non-parasitic redwings. To do so, we asked whether the direction of transcript expression differences in parasitic cowbirds as compared to adult red-winged blackbirds was in the same or opposite direction of that in juvenile red-winged blackbirds as compared to adult red-winged blackbirds. When differences between parasites and juveniles were in the same direction (*i.e.*, both up-regulated or down-regulated as compared to adult non-parasites) we considered transcripts as exhibiting juvenile-like expression. Conversely, when differences between parasites and juveniles were in opposite directions, we considered transcripts as exhibiting adult-like expression (Figure 4A). We performed this comparison only for those transcripts differentially expressed between parasites and adult non-parasites, as these represent the genes most likely involved in the transition to parasitic behavior. To test for significant neotenic expression, we asked whether the percentage of differentially expressed transcripts exhibiting juvenile-like expression was greater than that expected by chance. To do so we compared the percentage of differentially expressed transcripts exhibiting neotenic expression to the percentage of all transcripts exhibiting neotenic expression using chi-squared tests of independence in R (version 3.3.2; The R Foundation for Statistical Computing).

Finally, we explored in more detail the expression of candidate genes identified as playing a role in maternal and social behavior and categorized these genes as belonging to neuromodulators that regulate changes in existing cell function or structural genes that underlie large scale neural renovations. For this list of candidate genes, we used a less stringent p-value cutoff of < 0.1 after FDR correction as we identified these gene *a priori* and because there is a well-demonstrated issue with false negatives with FDR correction (Rice *et al.* 2008). Results of all DE comparisons are available on figshare 10.6084/m9.figshare.7098212.

### Data availability

RNA-seq data have been deposited in the NCBI Sequence Read Archive (Project #PRJNA523990 (brown heads), PRJNA523991 (bronzed), PRJNA523992 (red-wings)). Other data sets and code used in this study have been archived in Dryad (doi:10.5061/dryad.s36hm11).

## Results

### Overall characterization of transcript expression variation

**Figure 2:**
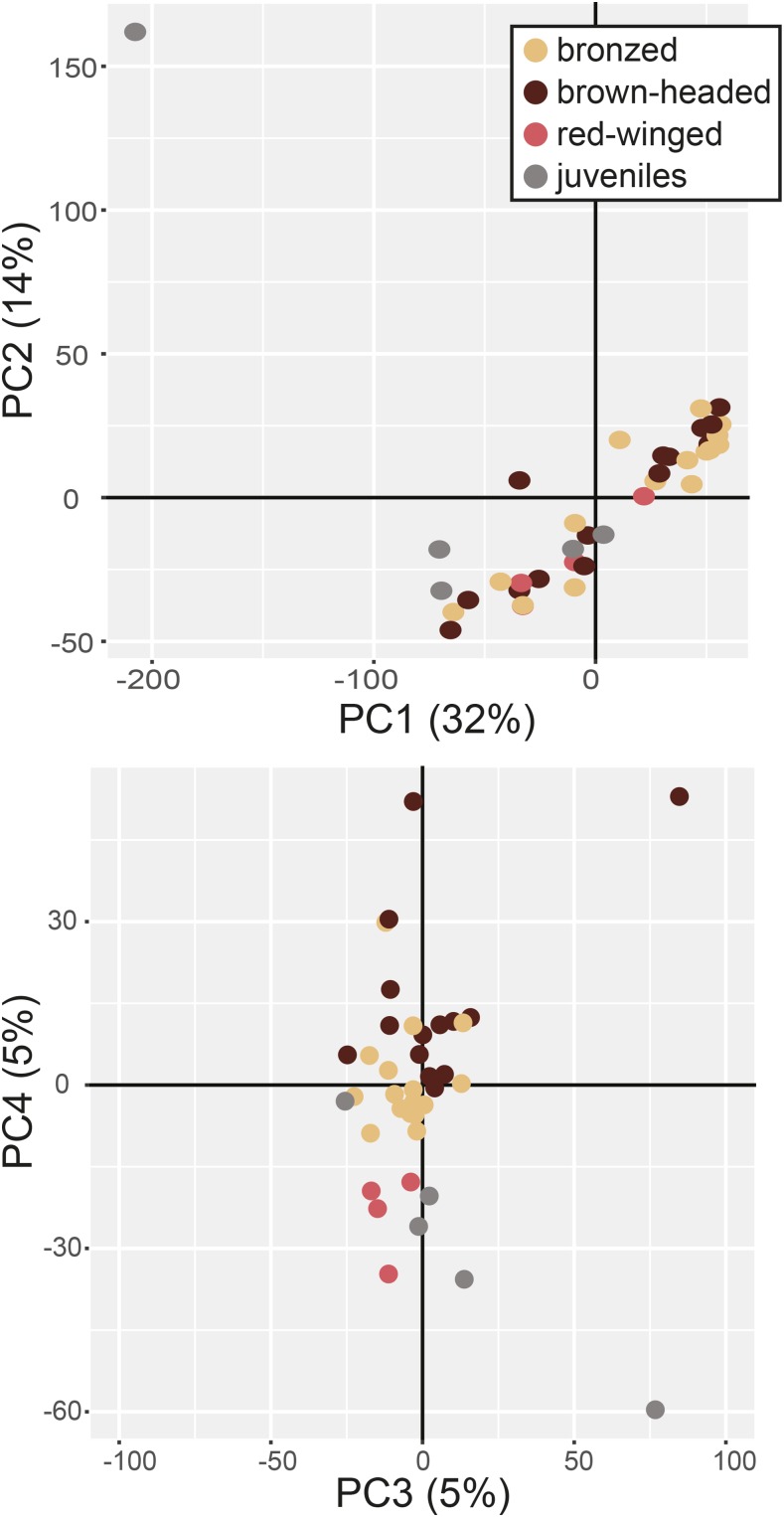
Principal components analysis of expression in all transcripts (12,237 orthologous) across all samples. Principal component 1 (PC1) explained 32% of the overall variation in gene expression and significantly separated adult parasitic bronze cowbirds (tan) and brown-headed cowbirds (brown) from juvenile (gray), but not adult (red), non-parasitic red-winged blackbirds. PC2 explained 14% of the overall variance in gene expression but was not associated with any experimental variables. PC3 and PC4 each explained 5% of the overall variance. While variation in PC3 was not associated with any experimental variables, PC4 separated parasitic bronzed and brown-headed cowbirds from non-parasitic red-winged blackbirds.

Prior to detailed differential expression analysis, we used principal component analysis to characterize expression variation across all transcripts (12,237 orthologous) and samples. Principal component 1 (PC1) explained 32% of the overall variation in gene expression and significantly separated adult parasitic bronze cowbirds and brown-headed cowbirds from juvenile non-parasitic red-winged blackbirds (F = 4.952, *P* = 0.0057; Figure 2). PC2 explained 14% of the overall variance in gene expression but was not associated with any experimental variables. PC3 and PC4 each explained 5% of the overall variance. While variation in PC3 was not associated with any experimental variables, PC4 separated parasitic bronzed and brown-headed cowbirds from non-parasitic red-winged blackbirds (F = 16.08, *P* < 0.0001; Figure 2).

### Differential expression between parasitic and non-parasitic adults

**Figure 3:**
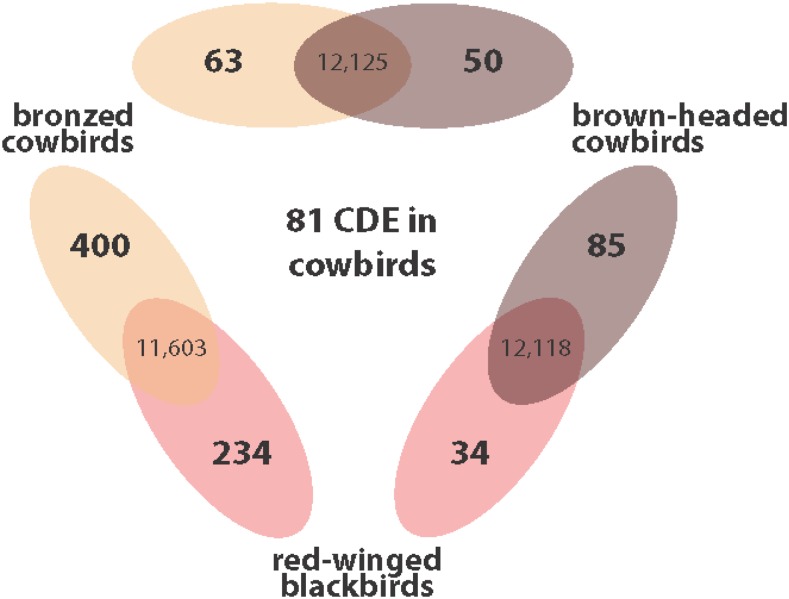
Differential gene expression comparisons between parasitic cowbirds and non-parasitic red-winged blackbirds. Transcripts differentially expressed in pairwise comparisons between species are shown in Venn diagrams with the number of differentially expressed transcripts on the edges and the number of non-differentially expressed transcripts in the overlap. The number of genes closer to a given species indicates those transcripts that were significantly upregulated in that species. Eighty-one transcripts were concordantly differentially expressed (CDE) in parasitic cowbirds as compared to non-parasitic red-winged blackbirds. We suggest these CDE transcripts are those transcripts most likely to be involved in the evolution of brood parasitism.

There were 119 differentially expressed transcripts between brown-headed cowbirds and red-winged blackbirds, 634 differentially expressed transcripts between bronzed cowbirds and red-winged blackbirds, and 112 differentially expressed transcripts between bronzed and brown-headed cowbirds (Figure 3; see Table S2 for complete list of DE genes; 10.6084/m9.figshare.7098143). Of the transcripts differentially expressed between parasites and adult red-winged blackbirds, 82 were overlapping between bronzed and brown-headed cowbirds. Of these 82 transcripts, 81 showed expression changes in the same direction in both parasites as compared to adult red-winged blackbirds. We refer to these 81 transcripts as concordantly differentially expressed (CDE; Figure 3, Table S2; 10.6084/m9.figshare.7098143). Changes in CDE genes in both parasitic cowbirds species as compared to non-parasitic red-winged blackbirds, suggest expression differences in these transcripts are those most likely associated with parasitic behavior.

We also performed GO-term enrichment analysis for all species comparisons and for the list of CDE genes (Table S3; 10.6084/m9.figshare.7098185). Notably, GO categories associated with neuropeptide signaling were significantly enriched in all comparisons among parasites and non-parasites, but not in comparisons between parasites (Table S3; 10.6084/m9.figshare. 7098185).

### Directionality of expression changes in parasites relative to non-parasites

Overall, transcript expression differences between parasites and red-winged blackbirds were not biased toward decreased expression in cowbirds relative to blackbirds: in brown-headed cowbirds 29% (85/119) of DE transcripts decreased expression relative to red-winged-blackbirds, and in bronzed cowbirds 37% (400/634) of DE transcripts decreased expression relative to red-winged-blackbirds. Of the CDE transcripts, 22% (63/81) decreased expression in both cowbirds relative to red-winged blackbirds (Figure 3; Table 1).

**Table 1:** Comparison of gene expression patterns to examine hypotheses of broad-scale downregulation and juvenile-like expression patterns in parasites as compared to non-parasites. DE = differentially expressed; CDE = concordantly differentially expressed; both = both brown-head and bronzed parasitic cowbirds

Comparison	# DE transcripts	downregulated	neotenic
bronzed *vs.* redwing	634	37%	74%
brown-head *vs.* redwing	119	29%	76%
both *vs.* redwing (CDE)	81	22%	78%

**Figure 4:**
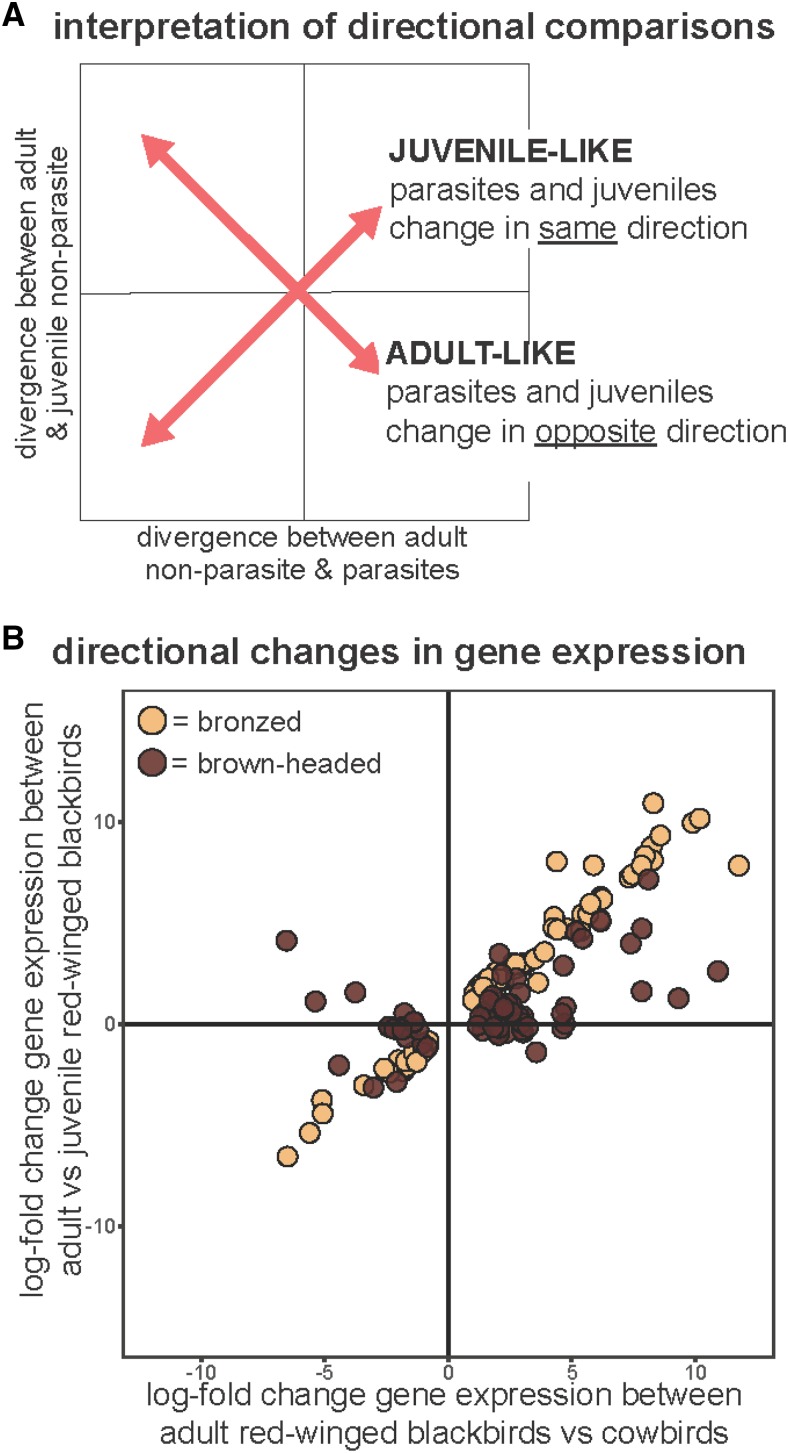
Neotenic patterns of gene expression in the POA of parasitic cowbirds. (A) We interpret gene expression changes in adult parasitic cowbirds as juvenile-like (neotenic) or adult-like compared to non-parasitic blackbirds based on the directional relationship between expression differences. When parasites mirror juveniles the relationship is positive (bottom left and top right quadrant) and when parasites mirror adults the relationship is negative (top left and bottom right quadrant). (B) We find that for both bronzed (tan circles) and brown-headed (brown circles) cowbirds expression patterns are overwhelmingly juvenile-like, with 78% percent of concordantly differentially expressed transcripts exhibiting juvenile-like expression (χ^2^ = 7.25, *P* = 0.0071).

To address the idea of juvenile-like transcript expression associated with a loss of maternal care, we additionally sampled juvenile red-winged blackbirds. We found only 16 genes differentially expressed between red-winged blackbird juveniles and adults (Table S4). While there were few significant expression differences, we were nonetheless able to use the direction of expression change in juveniles *vs.* adults to ask whether expression differences in cowbirds were more juvenile-like (neotenic) or adult-like. To do so, we compared the direction of transcript expression changes between parasites and adult non-parasites to that between juvenile and adult non-parasites. When differences in parasites and juveniles were in the same direction (*i.e.*, both up-regulated or down-regulated) as compared to adult non-parasites, we considered transcripts as exhibiting juvenile-like expression (Figure 4A). Conversely, when differences between parasites and juveniles were in opposite directions, we considered transcripts as exhibiting adult-like expression (Figure 4A).

Of those transcripts differentially expressed between bronzed cowbirds and red-winged blackbird adults, 74% showed juvenile-like expression in the parasitic species (χ^2^ = 47.869, *P* > 0.0001; Table 1). Similarly, 76% of transcripts differentially expressed between brown-headed cowbirds and adult red-winged blackbirds showed juvenile-like expression in the parasite (χ^2^ = 8.36, *P* = 0.0038; Table 1). Finally, for the transcripts concordantly differentially expressed (CDE) among bronzed and brown-headed cowbirds, 78% showed juvenile-like expression in the parasites (Figure 4B; χ^2^ = 7.25, *P* = 0.0071; Table 1). In brief, evolved expression differences between parasitic cowbirds and adult red-winged blackbirds overwhelmingly mirrored juvenile-like expression in the non-parasitic species.

### Expression differences in select social behavior-and maternal care-related candidate genes

**Figure 5:**
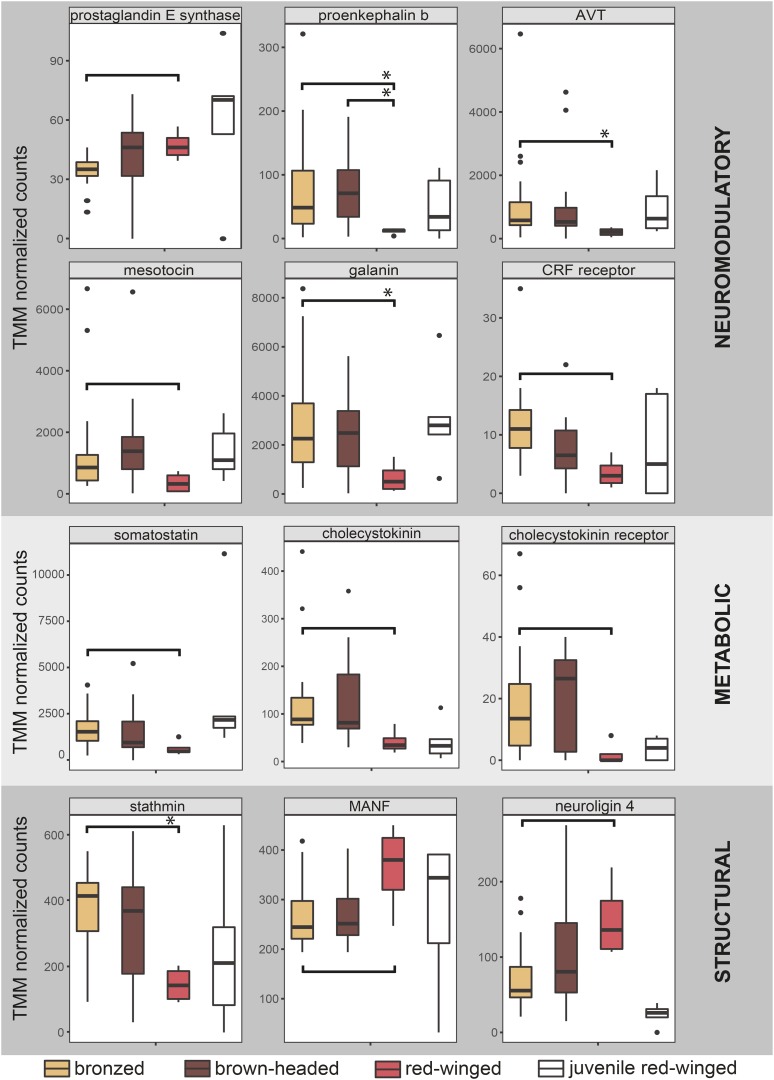
Transcript expression of candidate genes. Functional plasticity genes included neuromodulators and metabolic modulators. Structural genes included genes involved in changes to neural architecture. Select candidate genes known to be important in maternal and social behavior are presented here. Boxplots show TMM normalized transcripts counts for presumptive candidate genes in bronzed cowbirds (tan), brown-headed cowbirds (brown), adult red-blackbirds (pink), and juvenile red-winged blackbirds (white). Genes are grouped based on their known neuromodulatory (top), metabolic (middle), or structural (bottom) functions. Significant differences are indicated by brackets either with (*P* > 0.05) or without (*P* > 0.1) asterisks. Remaining differentially expressed candidate genes associated with structural and functional plasticity are listed in Table S4. AVT = arginine vasotocin; CRF receptor = corticotropin releasing factor receptor; MANF = mesencephalic astrocyte-derived neurotrophic factor.

Among transcripts differentially expressed between parasites and non-parasites, we identified twenty-two annotated as genes of interest with roles in structural and functional neural plasticity and/or metabolic regulation, twelve of which have known roles in social and maternal behavior (Figure 5; Hofmann and Fernald; 2000; Harris *et al.* 2005; Numan and Insel 2006; Trainor and Hofmann 2006; Martel *et al.* 2008; Jamain *et al.* 2008; Sakurai and Mieda 2011; Ey *et al.* 2012; Dulac *et al.* 2014; Wu *et al.* 2014; Weintraub *et al.* 1985; Bendesky *et al.* 2017). Again, 8 out of 12 of these candidate genes exhibited a juvenile-like expression pattern in parasitic cowbirds. Three of these maternal care-related candidate genes were significantly lower in the brood parasites compared to non-parasites, including prostaglandin E synthase, neuroligin-4 and mesencephalic astrocyte derived neurotrophic factor (Figure 5 and Table 2). One gene that may inhibit maternal care is elevated in brood parasites compared to non-parasites (corticotropin releasing hormone receptors (CRHR); Table 2). Table 2 lists six additional candidate genes with demonstrable roles in the regulation of social behavior but not maternal behavior.

**Table 2:** Differentially expressed genes that are candidate genes of interest based on known roles in maternal and social behavior. The +/− represents whether the regulatory pattern was higher or lower in the parasite compared to the non-parasite. Gene function is classified as structural (S), neuromodulatory (N), or metabolic (M). Citations for known roles in maternal and/or social behavior are provided

Candidate Gene	+ / -	Function	Citations
Acetylcholinesterase	+	N	Karvat & Kimchi (2014)
BDNF/NT-3 growth factors receptor	—	S	Branchi *et al.* (2006), Berry *et al.* (2015)
Early growth response protein 1	+	S	Lynch *et al.* (2017)
Gastrin-releasing peptide receptor	+	M	Yamada *et al.* (2000), Presti-Torres *et al.* (2007)
Orexigenic neuropeptide	+	M	Harris *et al.* (2005), Sakurai and Mieda (2011)
Somatostatin	+	N	Hofmann and Fernald (2000), Trainor and Hofmann (2006)

## Discussion

The evolution of avian brood parasitism provides a unique opportunity to investigate the mechanisms mediating parental care and its loss within a comparative framework. Here, we provide the first insights into the transcriptional mechanisms associated with the evolutionary transition from maternal care to brood parasitism in Icterids (blackbirds). Comparing POA-specific transcriptomes in parental and non-parental species, we identified both broad scale and transcript specific expression differences associated with parasitic behavior. While behavioral ecologists have provided many excellent explanations for an evolutionary loss of maternal care, the neurobiological basis of this behavior has been wholly unknown. This work provides the first insights into neurobiological and molecular bases for the loss of maternal behaviors in avian brood parasites.

### Expression differences associated with brood parasitism

Our first aim was to identify gene expression changes most likely involved in the transition to brood parasitic behavior. We used the power of our comparative approach to identify genes with significant, concordant expression differences in parasitic species as compared to the non-parasitic species. By focusing our attention on these overlapping, concordantly differentially expressed genes, we could most readily identify expression differences likely related to our shared phenotype of interest (*i.e.*, brood parasitism) rather than to non-shared species level differences. We identified 81 concordantly differentially expressed (CDE) transcripts, which we suggest represent the set of transcripts whose expression changes most likely contribute to the emergence of brood parasitic behavior. These transcripts provide excellent candidates for future functional studies and work in additional brood parasitic species and/or males brood parasites. Broader phylogenetic comparisons across genera will determine whether CDE transcripts identified here are implicated in convergent losses of maternal care across Passeriformes and provide insight into mechanisms of convergent behavioral evolution.

### Neotenic expression signatures

Our next aim was to identify broad scale patterns in differentially expressed transcripts. We focused on CDE transcripts because, as described above, we consider these as the genes most likely associated with a transition to brood parasitism. We predicted two non-mutually exclusive patterns could arise: downregulation of POA activity in parasitic species and/or juvenile-like expression in parasitic species as compared to maternal species. We did not find evidence for broad-scale transcript downregulation in parasitic species. While this does not preclude downregulation of key maternal care-related transcripts (see below) it suggests that brood parasitism does not arise from an overall decrease in POA transcriptional activity.

In contrast, clear evidence emerged for a shift toward juvenile-like (neotenic) expression in the POA of adult parasites. We note that this pattern was apparent in differentially expressed transcripts, despite the fact that principal component analysis of overall transcript expression most strongly separated adult parasites and non-parasitic juveniles. 78% of CDE transcripts exhibited juvenile-like expression in parasitic cowbirds, suggesting that the retention of a neotenic, non-maternal expression state in the POA may contribute to non-maternal, parasitic behavior. Neoteny in plumage color and skull ossification patterns has been described for brood parasitic birds (Payne 1967), so a compelling prediction is that this whole suite of neotenic traits are underpinned by broad-scale neoteny in gene expression.

Alterations of existing mechanisms through shifts in developmental timing have been linked to innovative morphologies and functionalities in other species (Shaw *et al.* 2007; Kur-Piotrowska *et al.* 2017), including behavioral traits in primates (Somel *et al.*, 2009). For example, Somel *et al.* (2009) demonstrated dramatic brain transcriptome remodeling in the human brain during postnatal development that was linked to delayed developmental changes relative to other primates. This transcriptional neoteny in humans was proposed to delay timing of human sexual development which, in turn, may have enhanced cognitive function in humans compared to other primates (Somel *et al.* 2009). From the current dataset, we cannot distinguish whether the neotenic patterns we observed represent a wholesale shift in expression timing across the brain of brood parasites, or differences in the extent and/or timing of delays are restricted to particular brain regions and molecular pathways. In either case, the patterns we report here suggest ontogenetic gene expression shifts in the POA have occurred in the evolution of avian brood parasitism. Building on this hypothesis, future studies will investigate neural and physiological patterns across development in brood parasites to determine the generality of a failure to transition from a juvenile into an adult-like state in driving brood parasitic behavior.

### Expression differences in social- and maternal-care related genes

To complement analysis of broad-scale expression patterns, we examined expression differences specifically in candidate genes know to be important in social and maternal behavior. Specifically, we asked whether expression patterns of candidate genes in parasitic species were consistent with downregulation of genes promoting maternal care or upregulation of genes inhibiting maternal care. Our examination of differentially expressed transcripts yielded candidate genes associated with structural and functional plasticity.

Neuromodulators such as mesotocin, AVT, and galanin have a clear and well-documented role in mammalian maternal behaviors (Numan and Insel 2006; Dulac *et al.* 2014; Bendesky *et al.* 2017). Prostaglandin facilitates both female reproductive behavior (Weintraub *et al.* 1985) and maternal behavior (Rosenblatt and Siegel 1981; Pedersen *et al.* 1982) in fish and mammals. In our study, prostaglandin E synthase was significantly downregulated in bronzed cowbirds as compared to the maternal species. This form of prostaglandin regulates female reproductive behavior in anurans (Weintraub *et al.* 1985). Additional candidate genes that were differentially expressed in one, but not both, parasitic species included mesencephalic astrocyte derived neurotrophic factor (MANF; Branchi *et al.* 2006; Figure 5) and neuroligin-4 (Jamain *et al.* 2008; Ey *et al.* 2012; Figure 5). Interestingly, the pattern of prolactin receptor expression in our study showed the same pattern reported by Ball (1991). Consequently, prolactin receptor, which is critical to the expression of maternal care in birds and mammals, is downregulated in the cowbird POA compared to the red-winged blackbird and this may facilitate the failure to transition into parental-like behaviors.

In addition to genes promoting maternal care, we also examined expression patterns in genes implicated in inhibiting maternal care in mammals. We identified corticotropin releasing hormone receptors (CRHR; Table 2) as significantly upregulated in bronzed cowbirds as compared to red-winged blackbirds. Studies in mice demonstrate that injections of corticotropin releasing hormone (CRH) inhibit maternal care and enhance aggression toward pups (Pedersen *et al.* 1991). However, our study identifies the receptors for CRH as being upregulated rather than CRH itself. An increase in CRF receptor expression may promote maternal care, rather than inhibit it. In mice, hippocampus-specific increases in receptors involved in the stress response axis result in decreased stress reactivity and increased maternal care (Liu *et al.* 1997). Thus, it is not clear whether POA-specific increases in CRHR may promote or inhibit maternal care in birds.

In contrast to genes related to functional plasticity, the role of structural plasticity related genes in parental and social behavior is less well established. For instance, stathmin is a phosphorylation-regulated tubulin-binding protein plays a critical role in regulating the microtubule cytoskeleton and may be required for axon formation during neurogenesis (Martel *et al.* 2008). Stathmin knockout mice have deficient innate and learned fear, which leads to inaccurate threat assessment. This, in turn, results in a profound loss in observed maternal behavior in females as they lack motivation for retrieving pups and are unable to choose a safe location for nest-building (Martel *et al.* 2008). While the functional outcomes of these modifications in transcript expression are not clear, these genes provide interesting candidates for future study on mechanisms of brood parasitism and general evolutionary shifts in parental behavior in both males and females.

While speculative, it is possible that experience-dependent brain plasticity is elevated during juvenile critical periods and declines into adulthood (Hübener and Bonhoeffer 2014; London *et al.* 2009). We suggest that the evolution of novel parasitic behaviors may be related to the ways in which peptides in our candidate gene list shape neural circuits and influence social processes during development. Heightened information storage during critical developmental periods in songbirds is associated with sustained gene expression, particularly immediate early genes, which may enhance sensitivity to song tutoring. In contrast, gene expression becomes suppressed in adults and is only induced when the adult bird experiences a salient social stimulus (Drnevich *et al.* 2012; Jin and Clayton 1997; London *et al.* 2009). Consequently, it is possible there may be a transferal from “constitutive plasticity” in juveniles to “regulated plasticity” in adults (London *et al.* 2009; Drnevich *et al.* 2012;), as has been proposed to explain song learning in birds (Jin and Clayton 1997). In other cases, critical developmental periods with elevated neural plasticity are mirrored by elevated mRNA expression patterns including corticotropin-releasing hormone (CRH; Romeo 2013). Thus, modification of neuromodulatory activity and structural plasticity across development provides a potential mechanism for the evolution of novel social behaviors across taxa.

### Summary

The evolution of avian brood parasitism provides fertile ground for the investigation of mechanisms mediating parental care in birds within a comparative framework. We provide the first insights into the neurobiological-basis of brood parasitism. We identify a set of genes whose expression changes are parallel across brood parasitic species and suggest that changes in these genes may also underlie transitions to brood parasitism in other lineages of parasites. In addition, we demonstrate patterns of juvenile-like transcript expression in the POA of parasitic species, providing a potential evolutionary and developmental mechanism by which brood parasitism could arise. Together, these results provide a foundation for future investigations of neural mechanisms mediating the emergence of alterative parental care strategies.
